# Unraveling age-related impairment of the neuromuscular system: exploring biomechanical and neurophysiological perspectives

**DOI:** 10.3389/fphys.2023.1194889

**Published:** 2023-06-22

**Authors:** M. Nùñez-Lisboa, M. Valero-Breton, A. H. Dewolf

**Affiliations:** ^1^ Laboratoire de Biomécanique et Physiologie et la Locomotion, Institute of Neuroscience, Louvain-la-Neuve, Belgium; ^2^ Exercise Science Laboratory, School of Kinesiology, Faculty of Medicine, Universidad Finis Terrae, Santiago, Chile

**Keywords:** neuromechanics, gait, physio-mechanics, direct current spinal stimulation, walking pattern

## Abstract

With extended life expectancy, the quality of life of elders is a priority. Loss of mobility, increased morbidity and risks of falls have dramatic individual and societal impacts. Here we consider the age-related modifications of gait, from a biomechanical and neurophysiological perspective. Among the many factors of frailty involved (e.g., metabolic, hormonal, immunological), loss of muscle strength and neurodegenerative changes inducing slower muscle contraction may play a key role. We highlight that the impact of the multifactorial age-related changes in the neuromuscular systems results in common features of gait in the immature gait of infants and older adults. Besides, we also consider the reversibility of age-related neuromuscular deterioration by, on the one hand, exercise training, and the other hand, novel techniques such as direct spinal stimulation (tsDCS).

## 1 Introduction

With extended life expectancy, the quality of life of elders is a priority. Loss of mobility, increased morbidity and risks of falls have dramatic individual and societal impacts. Among the many factors of frailty involved, loss of muscle mass and strength (Akima et al., 2001) and neurodegenerative changes (Rygiel et al., 2016) play a key role. Whether changes in the neural control precede or follow the decline of muscle mass and strength and how they both are related to gait alteration remains yet to be established. More than ever, this needs to be elucidated to implement interventions that can maintain or improve neuromuscular function in older adults.

Biomechanical changes with age have garnered considerable scientific attention for nearly 50 years. The scientific community (Winter et al., 1990; Delabastita et al., 2021) most often points to a reduction in mechanical power generated by the plantar flexor muscles during the push-off phase of walking as the hallmark biomechanical ageing features of gait. However, the 11%–35% decline in force or power-generating capacity of propulsive leg muscles cannot fully explain the age-related modification of gait in older adults. Indeed, (i) many old adults underutilize their available muscular capacity for generating propulsive power in walking and are able to increase it during slope walking or using biofeedbacks (Waanders et al., 2021), and (ii) age-related changes in kinematics have been found prior to the appearance of propulsion decline with increasing age (Sloot et al., 2021). Taken together, it suggests that the decline of propulsive power generation is thus not only due to a reduced muscular capacity, but neural factors are likely to contribute as well.

For instance, with aging motor weakness is due in part to neuromuscular degeneration, but also to degenerative changes in the central nervous system. Thus, reduction in grey matter volume (Good et al., 2001), number of motor cortical (Henderson et al., 1980) and spinal motor neurons (Doherty, 2003), synaptic density (Haug and Eggers, 1991), white matter integrity (Davis et al., 2009), and descending commands for motor activation (Yue et al., 1999) are some of the factors that may contribute to age-related motor impairment. Another determinant of functional capacity and autonomy is the integrity of other components of the neuromuscular system, which wires the brain and skeletal muscles via motor neurons and the neuromuscular junction. However, despite its obvious importance for rhythm generation, the potential involvement of the spinal cord in age-related modification of locomotion has received little attention.

Using an electrophysiological approach, a way to get insight into spinal cord functioning is to look at the spatiotemporal organization of the total locomotor output by mapping multi-muscles EMG onto the spinal cord in approximate rostro-caudal locations of the motoneuron (MN) pools (Ivanenko et al., 2008; Cappellini et al., 2010; Ivanenko et al., 2013; La Scaleia et al., 2014; Yokoyama et al., 2017; Dewolf et al., 2019a). By studying the spinal motor output across various walking conditions in older adults (walking at different speeds, backward, upslope, downslope, upstairs, downstairs), similar age-related differences in muscle activations have been observed despite the various biomechanical constraints (Dewolf et al., 2021a; Dewolf et al., 2021b). In particular, the activity profiles of the muscles innervated from the sacral segments were significantly wider in older adults in all conditions. Interestingly, similar modification has been observed in young children (Ivanenko et al., 2013; Dewolf et al., 2020b).

The major consideration of this review is the age-related remodeling of both the neural and muscular system and its relationship with locomotion changes with age, to shed light on the multifactorial age-related changes of gait. The alterations of the gait pattern in older adults are then compared to immature gait. Besides, we also consider the reversibility of age-related neuromuscular deterioration by, on the one hand, exercise training, and the other hand, novel techniques such as direct spinal stimulation (tsDCS) to mitigate the reduction of intrinsic spinal motoneuron excitability in older adults (Orssatto et al., 2021a), and how it could potentially lead to improved strategies for promoting locomotor function recovery.

## 2 Neuromuscular modification with aging

Aging is a natural and gradual process where the alterations in motor control and physical fitness are multifactorial (Borzuola et al., 2020). Strength capacity and muscle mass decrease during aging, in great part due to sarcopenia. Aging-related sarcopenia is the most common type of atrophy in humans. Specifically, sarcopenia is a progressive skeletal muscle disorder identified by low muscle strength, low muscle quantity or quality, and low physical performance (Cruz-Jentoft et al., 2019). It is associated with an increased risk of adverse outcomes, such as functional disability, poor quality of life, and a higher risk of mortality (Batsis et al., 2014; Beaudart et al., 2017; Kelley and Kelley, 2017; Liu et al., 2017). In older adults, the loss of strength seriously affects independence associated with activities of daily living but also leads to a greater risk of falls, which is strongly related to mortality (Suzuki et al., 1992; Landi et al., 2012). From a clinical perspective, it is essential to understand the mechanisms underlying the modifications in skeletal muscle morphology and function, which are evident during aging (Fiatarone et al., 1990). Muscle strength begins to decline after 30 years of age and continues to decline with advancing age (Gava et al., 2015). Changes related to muscle morphology and its electrophysiology generally appear after the age of ∼40, and it also continues to decrease progressively (Stålberg and Fawcett, 1982; Oertel, 1986; Murton, 2015). Therefore, changes in strength appear to precede changes associated with skeletal muscle morphology. Also, the effect of aging on skeletal muscles depends on muscle location and function, since leg muscles are more affected than arm muscles (Stålberg and Fawcett, 1982; Oertel, 1986).

Several age-related modifications of muscle tissue have been described, such as loss of muscle fibers (Lexell et al., 1983; Lexell, 1995; McPhee et al., 2018) or substantial loss of contractile proteins (Larsson et al., 1996), such as myosin heavy chain (Siparsky et al., 2014). Not only the reduction in the total number of fibres occurs, but also in their cross-sectional area (Lexell and Taylor, 1991). It should be noted that a differential response is reported in fibre loss depending on the type of muscle, with a faster decrease mainly observed in type II fibres (Cade and Yarasheski, 2006; Domingues-Faria et al., 2016). Also, a change from the fast myosin isoform to the slow isoform has also been observed, which has a lower capacity to generate force. This change in fibre type could contribute to both slowing movement and decreased maximal strength, and in turn induce age-related changes of gait.

Even the pathogenesis of sarcopenia is not yet fully understood, multiple etiological factors seem to be involved, including alteration of muscle proteostasis (Lecker et al., 2004), mitochondrial dysfunction and mitochondrial DNA deletions (Bua et al., 2006), deregulation of satellite cells (Shefer et al., 2006), and accumulation of extracellular matrix called fibrosis and fat infiltration into skeletal muscle (Song et al., 2004). The cause of sarcopenia cannot be solely attributed to alterations in skeletal muscles. In fact, the nerve responsible for stimulating muscle fibers plays a significant role in sarcopenia. As the skeletal muscles experience degeneration with age, the decline in neuromuscular function emerges as a crucial contributing factor (Aagaard et al., 2010). Evidence has shown alterations associated with a reduction in motor units. Studies have specifically compared the amount of motor neurons in young and elderly subjects, with the latter showing a 50% decrease (Doherty et al., 1993). In addition, the motor neurons begin to exhibit alterations in firing frequency and rate. The maximum firing frequency of motor neurons is lower compared to young subjects (Klass et al., 2008). Other changes have also been detected during aging related to a decrease in axonal conduction velocity, which is explained by reduced myelination and internodal length (Scaglioni et al., 2002). Therefore, evidence suggests that the compromised nervous system function may also be one of the important contributors to functional decline described in sarcopenia (Rygiel et al., 2016; Kwon and Yoon, 2017). Indeed, normal innervation and its corresponding regular activation are necessary to maintain muscle mass through muscle contraction. For example, there is an association between the loss of muscle fibres and the loss of motor units in older people (McNeil et al., 2005; Piasecki et al., 2016; Piasecki et al., 2018). In addition, slow motor neurons may be more adapted to reinnervation, leading to the loss of fast motor neurons with age. This could respond to the change in fibre type that occurs with aging (Larsson et al., 1978; Kadhiresan et al., 1996; Andersen, 2003).

While the causes of the age-associated loss of motor neurons are still unsettled, the neuromuscular junction integrity, and in particular the mitochondrial dysfunction at the neuromuscular junction, may have an important role (Shigemoto et al., 2010). The changes in the neuromuscular junction have been reported to be related to morphological alterations of the pre- and post-synaptic regions and to the reduction of synaptic vesicles (Jang and Van Remmen, 2011). The loss of motoneurons also plays an important role in the alterations of the excitation-contraction coupling process during aging (Payne and Delbono, 2004). Indeed, the decrease in isometric strength and contraction velocity appears before the reduction in muscle mass. Therefore, it has been proposed that the decrease in the number of motor units occurs before the loss of muscle function (Deschenes et al., 2010; Sheth et al., 2018). However, there is still insufficient evidence and more studies are required to complement the current hypothesis.

## 3 Gait during development and aging: a brief overview of the two sides of life

**FIGURE 1:**
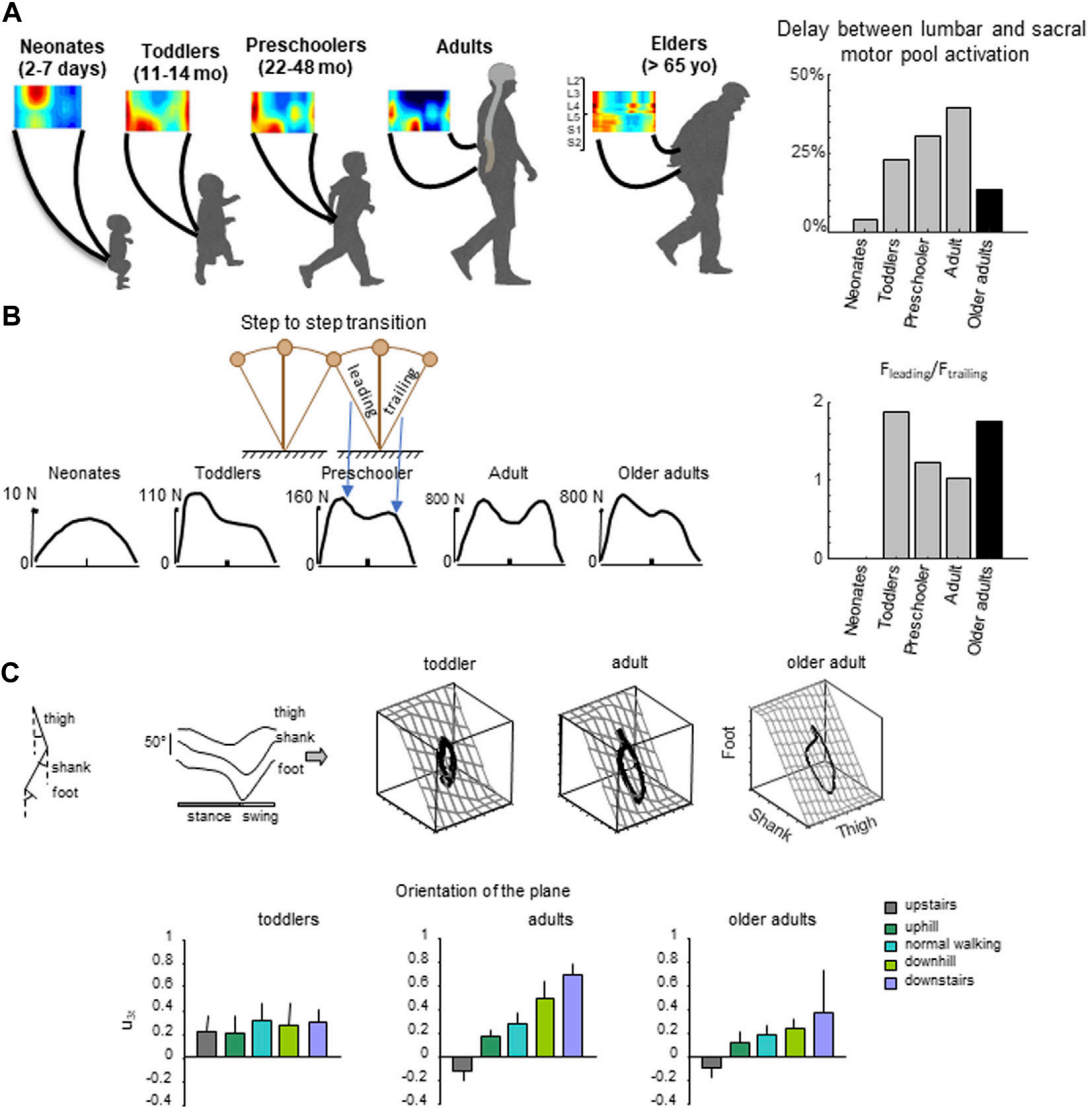
General features of gait in older adults. **(A)** spatiotemporal maps of motoneuron activity of the lumbosacral enlargement in neonates, toddlers, preschoolers, adults and older adults, and the delay between the activation of lumbar and sacral activation (data from Ivanenko et al. (2013) for neonates, toddlers, preschoolers and adults, and from Dewolf et al. (2021a) for older adults). **(B)** Schematic representation of walking like an inverted-pendulum. Below, representative vertical loading force during stepping in neonates, toddlers, preschoolers, adults and in older adults. The characteristic force profile was evaluated using the ratio between the leading limb and the tailing limb. **(C)** Intersegmental coordination assessed by principal component analysis (PCA) of limb segment elevation angles during walking. From left to right: thigh, shank, and foot elevation angles (relative to the vertical), corresponding 3D trajectory in segment angle space along with the interpolated plane (modified from Ivanenko et al. (2008)). Three examples of gait loops are presented (one toddler, one adult and one older adults). Below, changes in the orientation of the covariance plane during walking over different surfaces in toddlers, adults and older adults (modified from Dominici et al. (2010) and from Dewolf et al. (2021b)).

The multifactorial age-related changes in the neuromuscular system, summarized in the last section, are rather well documented. As people age, those changes result in alterations in gait patterns. In this section, we present different aspect of gait (presented in Figure 1) that are affected by age, and that interestingly resemble those seen in younger infants.

In humans, when EMG activity patterns are mapped onto the spinal cord in approximate rostrocaudal locations of the motoneuron (MN) pools, the activation of MNs tends to occur in bursts that can be associated with the major kinetic events of the gait cycle (Ivanenko et al., 2008; Cappellini et al., 2010; La Scaleia et al., 2014; Yokoyama et al., 2017; Dewolf et al., 2019a). In particular, the first burst occurs around foot contact and is mainly localized on the lumbar segment whereas the second burst occurs during the second part of the stance mainly and is localized on the sacral segment (Figure 1A). This approach provides information about pattern output in terms of lumbosacral segmental control (from L2 to S2) rather than in terms of individual muscle control.

In older adults, across different forms of walking gait, age-related differences were observed (Monaco et al., 2010; Santuz et al., 2020; Dewolf et al., 2021a; Dewolf et al., 2021b), suggesting specific adjustments of the pattern generation circuitries. In particular, the sacral output was significantly wider in older adults and occurred earlier in the stance (Figure 1A). Interestingly, this result does not simply reflect the documented distal-to-proximal modification of kinetics since the human spinal topography does not reflect the muscle topography on the lower limbs (Kendall, 2005). Instead, this potentially highlights a distal to proximal degeneration of the motor system. Accordingly, when the spinal excitability is estimated using the Hoffmann reflex technique, no difference is found between young and older adults on *vastus medialis* muscle (Mau-Moeller et al., 2013), whereas age-related modulations of the reflex response have been reported in *soleus* muscle (Baudry et al., 2015). Interestingly, while the craniocaudal gradient of corticospinal development in infancy is well established (Payne and Isaacs, 2017), less is known about the differential degeneration of different portions of the corticospinal tract with aging.

Development and aging can be seen as two opposite but complementary phenomena (Feltes et al., 2015). For example, it appears that projection tracts, such as the corticospinal tract, which develop earlier than association tracts in infancy, degenerate later than association tracts in older subjects. Also, primitive reflexes, which are commonly present in normal infants and disappear during development, reappear in patients with diseases of the nervous system but also in healthy older adults with an incidence increasing with age (Gossman and Jacobs, 1980; Jacobs and Gossman, 1980; Damasceno et al., 2005; van Boxtel et al., 2006; Hobo et al., 2014). Indeed, attempts to elicit primitive reflexes are a routine part of the standard neurological examination in the elderly, with the following reflexes tested: *e.g.*, snout, suck, palmomenral, and hand grasp (described in detail by Koller (Koller, 1984)). Another reflex observed at the beginning of life is the stepping reflex: human newborns step on the ground if supported (Thelen and Fisher, 1982; Forssberg, 1985; Yang et al., 1998; Dominici et al., 2011; Dewolf et al., 2020b; Dewolf et al., 2020a), and stepping generally disappears a few weeks after birth unless trained. The relationship between this reflex and mature walking gait has been argued (Andre-Thomas and Autgaerden, 1966; Dominici et al., 2011; Sylos-Labini et al., 2022), with lower ow complexity and higher variability of neuromuscular signals in neonates. Because of the less complex and more variable control of muscle in older adults (Allen and Franz, 2018), and based on the common features of gait between infants and older adult presented in the present section (Figure 1), One may speculate about the potential greater similarities between neonatal stepping and older adult’s gait pattern.

Also from a kinematic point of view, a simpler coordination pattern among the lower limb segments can be observed both during childhood and agedness (Ivanenko et al., 2004; Noble and Prentice, 2008; Dominici et al., 2010; Bleyenheuft and Detrembleur, 2012; Dewolf et al., 2019b; Gueugnon et al., 2019). One way to unravel the multi-segmental coordinative law is the so-called coplanar variation (Borghese et al., 1996; Bianchi et al., 1998b). During walking, each lower-limb segment oscillates back and forth relative to the vertical with a similar waveform, time-shifted across different segments (Figure 1C). The lower limb segment angles do not evolve independently of each other, but they are tightly coupled: when plotted one vs. the others, they co-vary along a plane, describing a characteristic loop over each stride (Figure 1C). The specific shape and orientation of the plane reflects the phase relationship between segments and therefore the timing of the intersegmental coordination (Barliya et al., 2009). Even if the intersegmental coordination in toddlers rapidly evolves toward the adult shape with experience (Cheron et al., 2001; Ivanenko et al., 2004; Dominici et al., 2010), when toddlers step in various conditions (slope, stairs, backward), they do not adapt their segmental coordination as adults do. Instead, they keep constant phase relationships (Dominici et al., 2010) (Figure 1C). In older adults, the modification of plane orientation across gait conditions is less adapted than in young adults (Dewolf et al., 2019b; Dewolf et al., 2021a; Dewolf et al., 2021b). Since the changes in planar covariation are thought to reflect the ability to adapt to different gait conditions (Bianchi et al., 1998b; Martino et al., 2014; Dewolf et al., 2018), the lack of changes observed in toddlers and to a lesser extent in older adults suggest reduced ability to adapt gait to environment or specific constraits (Dominici et al., 2010).

Because a link between center of mass (COM) trajectory and functional spinal cord topography has been previously highlighted (Cappellini et al., 2010; Dewolf et al., 2019a; Dewolf et al., 2020b), one may expect comparable COM dynamics in older adults and young infants. Center of mass (COM) mechanics is a fundamental concept in biomechanics that describes the movement and balance of an individual’s body. In young adults, during walking the COM vaults over a relatively stiff limb with the heel well in front of the hip at the beginning of the stance, and the heel lift with maintained toe contact at the end of the stance. One of the direct consequences of such a heel-to-toe roll-over pattern is that the extension of distal joints is delayed relative to proximal joints, leading to the typical double hump shape (so-called ≪m − pattern≫) of the vertical ground reaction force (Hallemans et al., 2006) (Figure 1A). In both older adults and younger infants, the walking gait lacks the specific m-pattern shape of adult heel-to-toe roll-over walking pattern (Forssberg, 1985; Dominici et al., 2011; Sylos-Labini et al., 2017), due to the lack of late push-off from the trailing leg (Dominici et al., 2011, 201; Dewolf et al., 2019b; Gueugnon et al., 2019). Another similarity is that both young infants and older adults have limited control over their COM (Foster et al., 2019; Malloggi et al., 2019). In the next section, we discussed the potential cause of modification of gait in older adults, resulting in kinematics, kinetics and neural similarities with the gait observed in children.

## 4 Could we counteract the age-related modification of gait?

Based on the well-documented change in muscle strength with aging described in the last section, a lot of efforts have been made to counteract it using exercise (Christie, 2011; Hortobágyi et al., 2015; Keating et al., 2021; Wu et al., 2021). Physical training is reported as an effective treatment for maintaining muscular function (Kraemer et al., 2002; Chen et al., 2021; el Hadouchi et al., 2022; Markov et al., 2022). Guidelines recommend high physical activity levels to increase health benefits in older adults (Boyer et al., 2012; Taylor, 2014), since it is supposed to enhance daily activities like gait. Resistance or power training not only increases/maintains muscle mass, strength, power and functional capacity in older adults, but it also induces several neuromuscular adaptations, such as an increase in peak firing frequencies of motoneurons. Also, older adults still practicing long-distance running reduce the decline in muscle function with age by enhancing the neural drive to the muscle (Cogliati et al., 2020).

Resistance training (RT) positively affects walking speed (Keating et al., 2021). For example, Hortobágyi et al. (2015) found that RT significantly increases the habitual gait speed of healthy old adults by 8.4% as a long-term effect. Power training also impacts gait velocity (Beijersbergen et al., 2017a; Beijersbergen et al., 2017b; Beijersbergen et al., 2017c), changing the rate of force development, which is moderately correlated with gait speed (Stock et al., 2019), and improving the functional performance (Radaelli et al., 2023). The main related effect of muscular training is a higher ‘habitual walking speed’ after the exercise sessions. However, training fails to directly translate to improved propulsive power generation in walking (Beijersbergen et al., 2013). For example, a higher level of physical activity in older adults did not mitigate the age-related modification of kinematic coordination and distal-to-proximal redistribution (Boyer et al., 2012). Also, greater muscular power (more than muscle strength) has been reported to have a strong influence on mobility (Bean et al., 2003), but without a clear change in gait pattern. Indeed, the biomechanical, physiological, and motor control adaptations in gait with training are still unknown, and physical trainers or physiotherapists lack consistent biomechanical data to understand the adaptation mechanism (Beijersbergen et al., 2017c). Based on lower limb coordination, Bianchi et al. (Bianchi et al., 1998a) showed that trained young subjects can exploit better the dynamic coupling between segments to save mechanical energy than untrained young subjects. It is, therefore, plausible that training in older adults may affect the lower limb intersegmental coordination to allow optimised gait mechanics.

While walking speed has been suggested to predict frailty and disability in older adults (Guralnik et al., 2000), we believe that evaluation of spontaneous walking speed is not the best outcome to evaluate the age-related decline of gait. Spontaneous gait speed, if not performed after period of familiarization sessions and following standardized instructions, may vary with the mood, motivation, stimuli of the experimenters, *etc.* For example, in a classical paper, Bornstein and Borstein (Bornstein and Bornstein, 1976) showed that the ‘pace of life’, measured as the spontaneous speed, varies with the size of the local population, regardless of the cultural setting, suggesting that immediate social and physical environment exert strong control over individual habitual speed (Levine and Norenzayan, 1999). Therefore, we believe that there’s an imperative need to understand the role of physical exercise in the process of age-related modification of neuromuscular control of gait. In particular, the data to interpret the mechanism needs to be more quantitative.

Enhancing physical capacity alone may not be sufficient to mitigate the age-related decline of the neuro-muscular system, such as the distal to proximal degeneration of the motor system highlighted above. A recent rehabilitation approach is the use of real-time biofeedback to encourage favorable biomechanical adaptations. For example, it has been showed that the propulsive power can be increased during walking in older adults using ankle power biofeedback (Browne and Franz, 2019), resulting in a reduction of distal-to-proximal redistribution of joint efforts. Based on the age-related modification of gait highlighted in Figure 1, one may expect that other parameters, such as the center of mass trajectory, can be manipulated using real-time biofeedbacks (e.g., as in Massaad et al., 2007). In particular, using real-time biofeedback to cue an acute change in the peak of vertical ground reaction force or in the limb loading may be an effective gait training intervention to mitigate the effect of age in older adults. Such approach has been developed in gait retraining following anterior cruciate ligament reconstruction (Luc-Harkey et al., 2018; Armitano-Lago et al., 2020) but not, to the best of our knowledge, with older adults.

Also, not only muscles but also the firing characteristics of our spinal motoneurons play a critical role in producing force, and so, performing daily activities. Motoneuron firing is determined by complex factors, such as ionotropic synaptic input and persistent inward currents (PICs) (Orssatto et al., 2021b). PICs are depolarizing currents generated by voltage-sensitive sodium and calcium channels. Hassan et al. (Hassan et al., 2021) (2021) found weaker estimates of PICs in older adults than in their younger counterparts, and propose that this weakening is an underlying mechanism for the slowing of motoneuron firing with ageing. Interestingly, the similarities observed between infants’ and older adults’ locomotor patterns (Figure 1) might be related to the slower and weaker firing characteristics (Dayanidhi et al., 2013).

As described by Hassan et al. (Hassan et al., 2021), the PICs weakening might result from a multitude of factors: (1) deterioration within the monoaminergic systems, (2) imbalance between excitatory and inhibitory synaptic inputs, or (3) changes in the function of monoaminergic receptors or voltage-gated channels. The question that need to be answered is now: how can we counteract the age-related decline in PICs? Our proposed answer for a future research question in this context is spinal neuromodulation, a promising strategy to augment spinal cord activity. In particular, non-invasive trans-spinal cord direct current stimulation (tsDCS) may improve spinal motor circuit function and motor output (Jankowska, 2017; Song and Martin, 2017) in older adults, because of the increase in firing frequencies of motoneuron (Bączyk et al., 2019) its specific effect of augmenting PIC-like responses induced by c-tsDCS, L-type Ca2C channel activation (Song and Martin, 2022).

The tsDCS has been increasingly used over recent years in the rehabilitation of patients following neurological injuries (Levins and Moritz, 2017; Gad et al., 2021; Taylor et al., 2021) or as an addition to physical training in sports (Berry et al., 2017). The effect of tsDCS on the gait patterns of older adults has not been studied yet. However, enhancing the PIC-like response of motor units would be well-suited to mitigate the effect of aging on spinal motor output. We hope that the ideas presented here help to motivate future efforts in understanding the quantitative modification of gait with aging and in evaluating a promising method that could be used as a supplementary tool in the management of geriatric patients.

## 5 Concluding remarks

This review outlines great similarities between the ‘first steps’ of infants and the ‘last steps’ of older adults. While part of the modifications observed in older adults may emerge from a lack of propulsive power, other neurodegenerative changes play a key role. In particular, slower muscle contraction is observed, resulting from the change in fiber type, the greater reinnervation of slow motor neurons or the lower motoneuron firing frequency with ageing, which is also an important peripheral contributor to the lack of adult-like locomotor patterns in early infancy (Dewolf et al., 2020b). Gaining insights into the age-related changes in human gaits may provide important clinical implications. For instance, we propose a novel intervention to enhance the PIC-like response of the motor unit, and in turn, mitigate the effect of aging.
